# Changes in The Trends of Tuberculosis-related Indicators in Hamadan Province Using the Join Point Regression Approach From 2011 to 2022

**DOI:** 10.34172/jrhs.2025.176

**Published:** 2024-12-25

**Authors:** Faezeh Ghasemi, Jalal Poorolajal, Salman Khazaei, Ali Zahiri, Fatemeh Torkaman Asadi

**Affiliations:** ^1^Department of Epidemiology, School of Public Health, Hamadan University of Medical Sciences, Hamadan, Iran; ^2^Student Research Committee, Hamadan University of Medical Sciences, Hamadan, Iran; ^3^Modeling of Noncommunicable Diseases Research Center, Hamadan University of Medical Sciences, Hamadan, Iran; ^4^Research Center for Health Sciences, Hamadan University of Medical Sciences, Hamadan, Iran; ^5^Deputy of Health, Hamadan University of Medical Sciences, Hamadan, Iran; ^6^Infectious Disease Research Center, Hamadan University of Medical Sciences, Hamadan, Iran

**Keywords:** Trend, Tuberculosis, Joinpoint, Incidence, Prevalence, Indicators

## Abstract

**Background:** This study was conducted to investigate the trend of some tuberculosis (TB) indices and identify existing gaps in addressing this important public health issue in Hamadan province over a long time period.

**Study Design:** A registry-based cross-sectional study.

**Methods:** In this study, we examined the trend of 10 TB indicators separately in males and females, including the incidence rates of smear-positive pulmonary TB (SPPT), extra-pulmonary TB (EPT), and smear-negative pulmonary TB (SNPT), co-infection with AIDS, relapse rate, smear conversion rate two months after treatment initiation, TB mortality rate, diagnosis rate of pulmonary TB with a smear grade of 3+, treatment success rate, and TB diagnosis rate by the private sector in Hamadan province during 2011-2022. The trend analysis of TB was conducted using Joinpoint regression model, and the annual percentage change (APC) and the average annual percentage change (AAPC) were calculated.

**Results:** A total of 481 females and 554 males were eligible for analysis. The incidence of SPPT in females showed a decreasing trend (AAPC: -7.72; 95% CI: -15.63, -1.10; *P*=0.008). The rates of EPT and treatment success showed a significant downward trend in both genders. In contrast, the recurrence rate among females exhibited a notable upward trend during the specified time period (AAPC: 18.45; 95% CI: 3.23, 46.47; *P*=0.0002).

**Conclusion:** The findings of this study suggest that the epidemiological profile of TB has exhibited a relatively favorable trend in some of the examined indicators since 2011, with declines observed in both SPPT and EPT.

## Background

 Tuberculosis (TB) remains one of the primary causes of illness and death in developing countries, especially among individuals with weakened immune systems such as people living with HIV (human immunodeficiency virus), malnutrition, or diabetes, as well as those who use tobacco.^1^ It is estimated that TB caused approximately 1.5 million deaths in 2019, with annual TB cases numbering around 10 million. TB remains a public health challenge globally and in developing countries, including Iran, it ranks ninth among the global burden of diseases.^2^ TB ranks thirteenth among the leading causes of death globally, and it is also the leading cause of death from a single infectious agent, posing a considerable concern for public health and a global public health priority.^3,4^ The United Nations’ Sustainable Development Goals and the WHO’s End TB Strategy aim to end the global TB epidemic by reducing TB-related deaths by 95% and reducing new cases by 90% by the year 2035.^5^

 In Iran, TB is a public health concern and a challenging healthcare issue due to various factors such as migration, poor economic conditions, and comorbidities like diabetes and HIV/AIDS, all contributing to an increasing incidence rate.^6,7^ Success in any healthcare program depends on monitoring the epidemiology of diseases and identifying gaps. Despite successful treatment of TB and a low number of multi-drug resistant cases, the incidence of TB in Iran has not significantly decreased.^8^

 Trend analysis plays a crucial role in understanding the dynamics of disease patterns over time. By examining the trends of TB-related indicators, we can gain valuable insights into the effectiveness of TB control measures and identify areas for improvement. This information is vital for optimizing resource allocation and achieving the best outcomes in TB reduction and prevention. Despite the importance of TB epidemiology, comprehensive studies estimating its burden are limited, particularly in developing countries such as Iran. Previous studies have often focused on a narrow range of indicators and have seldom utilized advanced analytical methods. Therefore, in this study, we aimed to fill this gap by conducting a detailed trend analysis of a wide range of TB-related indicators using the joinpoint regression model.

## Materials and Methods

 In the present registry-based cross-sectional study, all TB cases in Hamadan province from 2011 to 2022- were investigated. In this study, the researchers obtained data from the TB registry, which collects information on TB patients from all districts periodically. The TB data are gathered by TB experts at the district health centers, evaluated for accuracy and quality, and then transferred to the provincial Deputy of Health. Therefore, information from different counties is aggregated. Patients with incorrect diagnoses or those who were imported during the treatment were excluded from our analysis. We examined 10 indicators, separately in males and females, including the incidence rate of smear-positive pulmonary TB (SPPT), the incidence rate of extra-pulmonary TB (EPT), the incidence rate of smear-negative pulmonary TB (SNPT), the co-infection of TB and AIDS, the relapse rate, the smear conversion rate two months after the treatment initiation, the mortality rate due to TB, the diagnosis rate of pulmonary TB with a smear grade of 3 + , the treatment success rate, and the TB diagnosis rate by the private sector. We calculated these indicators primarily in this study based on individual-level data. The formulas for estimating the specified indicators are presented in Table S1 (Supplementary file 1).

 According to national guidelines, the diagnostic criteria for pulmonary TB were at least two positive sputum smears out of three consecutive sputum samples, a positive smear in one sample along with radiographic evidence of TB disease, or a positive smear in one sample along with a positive sputum culture.

 The amount of change in the investigated indicators between time intervals t and t + 1 is estimated using linear regression on the natural logarithm of annual rates and using the calendar year as the predictor variable.


$$ln\left(r_{t}\right)=b_{0}+b\times t$$

In which ln(r) represents the natural logarithm of the rate in year *t*. Then, the annual percentage change (APC) from year t to *t* + 1 is calculated as follows:


$$\frac{r_{t+1}-r_{t}}{r_{t}}*100$$

$$\frac{e^{b_{0}+b\left(t+1\right)}-e^{b_{0}+b*t}}{e^{b_{0}+b*t}}*100$$

$$APC=\left(e^{b}-1\right)*100$$

To calculate the APC, we only need one b. The average annual percentage change (AAPC) is calculated using the following formula:


$$AAPC=\left\{\left\{Exp\left(\frac{\sum w_{j}b_{j}}{\sum w_{j}}\right)-1\right\}\times 100\right.$$

Where b_i_s are the slope coefficients for each segment in the desired range of years, and the w_i_s are the length of each segment runs in the range of years. A positive APC indicates an increase in the measured variable during the specified period, while a negative APC indicates a decrease in that variable over the same timeframe. The numerical value of APC reflects the annual rate of change. Similarly, a positive AAPC denotes an overall increase in the variable over a specified period of time, whereas a negative AAPC indicates an overall decrease. The numerical value of AAPC serves as a summary measure of the trend over multiple time points, capturing variations in trends throughout the period.

 The trend analysis of TB incidence or mortality was conducted using the Joinpoint regression model. This method is utilized to identify short-term changes in the trend. The permutation method was employed to find additional joinpoints in describing the existing trend. Finally, the optimal number of joinpoints for describing the trend was selected. The percentage change in annual rates was calculated using logarithmic linear regression, with the calendar year as the predictor variable. When no joinpoint exists (i.e., no change in the trend), the APC remains constant and equals the AAPC. Otherwise, the entire period is divided by points with changing trends. Then, the AAPC is estimated as the weighted average of the estimated APC in each segment using the segment length as the weight. When the number of joinpoints (k) is determined, different models with k joinpoints were compared using the Bayesian information criterion (BIC). Data analysis was performed using the Joinpoint regression program. A significance level of 0.05 was considered for the statistical tests.

## Results

 In this study, we analyzed data from the TB registry, which included information on 481 females and 554 males registered in Hamadan Province from 2011 to 2022. The frequency and incidence of investigated indices in both males and females during the mentioned time period are presented in Tables 1 and 2.

**Table 1:** Incidence rate of some TB indicators in Hamadan province from 2011 to 2022

**Years**	**The incidence of SPPT**^a^		**The incidence rate of EPT**^a^		**The incidence rate of SNPT**^a^		**The negative rate of sputum smear two months after the start of treatment**^b^		**The detection rate of SPPT with a result of 3+**^a^		**Co-infection rate of SPPT and AIDS**^a^	
	**Frequency**	**Incidence**	**Frequency**	**Incidence**	**Frequency**	**Incidence**	**Frequency**	**Incidence**	**Frequency**	**Incidence**	**Frequency**	**Incidence**
**Male**												
2011	22	2.37	11	1.19	6	0.64	9	40.90	12	54.54	1	4.54
2012	22	2.35	15	1.60	12	1.28	16	72.72	7	31.81	1	4.54
2013	33	3.49	15	1.58	12	1.26	24	72.72	14	42.42	2	6.06
2014	17	1.78	12	1.26	11	1.15	7	41.17	8	47.05	2	11.76
2015	27	2.94	9	0.98	4	0.43	17	62.96	12	44.44	6	22.22
2016	31	3.34	23	2.48	12	1.29	9	29.03	12	38.70	0	0
2017	27	3.06	19	2.15	13	1.47	11	40.74	10	37.03	1	3.70
2018	20	2.43	5	0.61	6	0.72	10	50.00	8	40.00	1	5
2019	22	2.75	16	2.00	4	0.50	10	45.45	8	36.36	0	0
2020	10	1.24	7	0.87	4	0.49	9	90.00	5	50.00	0	0
2021	15	1.84	7	0.86	3	0.36	5	33.33	13	86.66	0	0
2022	17	2.04	7	0.84	4	0.47	1	5.88	9	52.94	0	0
**Female**												
2011	33	3.76	16	1.82	3	0.34	27	81.81	11	33.33	0	0
2012	27	3.05	24	2.71	3	0.33	19	70.37	9	33.33	1	3.70
2013	30	3.73	22	2.74	7	0.87	23	76.66	9	30.00	1	3.33
2014	21	2.32	13	1.43	2	0.22	15	71.42	8	38.00	1	4.76
2015	23	2.63	11	1.26	7	0.79	16	69.56	7	30.43	0	0
2016	32	3.62	16	1.81	6	0.67	15	46.56	9	28.12	0	0
2017	18	2.14	16	1.91	8	0.95	7	38.88	11	61.11	0	0
2018	11	1.40	9	1.14	6	0.76	6	54.54	6	54.54	0	0
2019	19	2.46	11	1.42	2	0.25	8	42.10	8	42.10	0	0
2020	6	0.77	7	0.90	4	0.51	3	50.00	1	16.66	0	0
2021	17	2.15	5	0.63	1	0.12	6	35.29	6	35.29	2	0
2022	10	1.23	7	0.86	2	0.24	4	40.00	4	40.00	0	0
**All**												
2011	55	3.05	27	1.50	9	0.49	36	65.45	23	41.81	1	1.81
2012	49	2.69	39	2.14	15	0.82	35	71.42	16	32.65	2	4.08
2013	63	3.08	37	1.81	19	0.93	47	74.60	23	36.50	3	4.76
2014	38	2.04	25	1.34	13	0.69	22	57.89	16	42.10	3	7.89
2015	50	2.79	20	1.11	11	0.61	33	66.00	19	38.00	6	12.00
2016	63	3.48	39	2.15	18	0.99	24	38.09	21	33.33	0	0
2017	45	2.61	35	2.03	21	1.21	18	40.00	21	46.66	1	2.22
2018	31	1.92	14	0.87	12	0.74	16	51.61	14	45.16	1	3.22
2019	41	2.61	27	1.72	6	0.38	18	43.90	16	39.02	0	0
2020	16	1.01	14	0.88	8	0.50	12	75.00	6	37.50	0	0
2021	32	1.99	12	0.75	4	0.24	11	34.37	19	59.37	0	0
2022	27	1.64	14	0.85	6	0.36	5	18.51	13	48.14	0	0

SPPT: Smear-positive pulmonary tuberculosis, EPT: Extra pulmonary tuberculosis, SNPT: Smear-negative pulmonary tuberculosis. ^a^ Per 100 000 population; ^b^ Per 100 SPPT patients.

**Table 2:** Incidence rate of some TB-related indicators in Hamadan province from 2011 to 2022

**Years**	**Treatment success rate**^a^		**TB detection rate by the private sector**^b^		**Recurrence rate**^a^		**The death rate from TB**^b^	
	**Frequency**	**Incidence**	**Frequency**	**Incidence**	**Frequency**	**Incidence**	**Frequency**	**Incidence**
**Male**								
2011	15	68.18	5	12.82	4	18.18	4	10.25
2012	20	90.90	13	26.53	0	0	5	10.20
2013	26	78.78	14	23.33	2	6.06	5	8.33
2014	11	64.70	8	20.51	2	11.76	8	20.51
2015	17	62.96	5	12.50	3	11.11	10	20.00
2016	16	51.61	7	10.60	2	6.45	12	18.18
2017	16	59.25	8	13.55	1	3.70	14	23.72
2018	16	80.00	3	9.37	2	10.00	1	3.12
2019	11	50.00	12	27.90	0	0	11	25.58
2020	7	70.00	7	33.33	3	30.00	1	4.76
2021	9	60.00	6	24.00	1	6.66	3	12.00
2022	6	35.29	1	3.57	2	11.76	4	14.28
**Female**								
2011	29	87.87	14	26.41	0	0	3	5.66
2012	21	77.77	14	25.92	0	0	4	7.40
2013	27	90.00	15	25.00	1	3.33	2	3.33
2014	19	90.47	8	22.22	1	4.76	2	5.55
2015	18	78.26	4	9.75	1	4.34	5	12.19
2016	27	84.37	8	14.81	1	3.12	4	7.40
2017	12	66.66	13	30.95	1	5.55	6	14.28
2018	9	81.81	4	14.81	2	18.18	4	14.81
2019	11	57.89	7	21.87	2	10.52	9	28.12
2020	5	83.33	7	41.17	4	66.66	1	5.88
2021	10	58.82	4	17.39	0	0	4	17.39
2022	4	40.00	4	21.05	1	10	0.5	0
**All**								
2011	44	80.00	19	20.65	4	7.27	7	7.60
2012	41	83.67	27	26.21	0	0	9	8.73
2013	53	84.12	29	24.16	3	4.76	7	5.83
2014	30	78.94	16	21.33	3	7.89	10	13.33
2015	35	70.00	9	11.11	4	7.84	15	18.51
2016	43	68.25	15	12.50	3	4.76	16	13.33
2017	28	62.22	21	20.79	2	4.44	20	19.80
2018	25	80.64	7	11.86	4	12.90	5	8.47
2019	22	53.65	19	25.33	2	4.87	20	26.66
2020	12	75.00	14	36.84	7	43.75	2	5.26
2021	19	59.37	10	20.83	1	3.12	7	14.58
2022	10	37.03	5	10.63	3	11.11	4	8.51

^a^ Per 100 SPPT patients; ^b^ Per 100 all TB types.

 In all indicators, except for “the death rate from TB” in males (39.29%) and “the detection rate of SPPT with a smear grade of 3 + ” in females (20%), a decreasing trend was observed in “the incidence of SPPT” in males (-29.41%) and females (-52.57%), “the incidence rate of EPT” in males (-29.41%) and females (-52.57%), “the incidence rate of SNPT” in males (-26.52%) and females (-29.41%), “the negative rate of sputum smear two months after the treatment initiation” in males (-85.64%) and females (-51.16%), “the detection rate of SPPT with a smear grade of 3 + ” in males (-29.93%), “treatment success rate” in males (-48.25%) and females (-54.49%), “TB detection rate by the private sector” in males (-72.23%) and females (-20.30%), and “recurrence rate” in males (-35.29%) (Tables 1 and 2).

**Figure 1:**
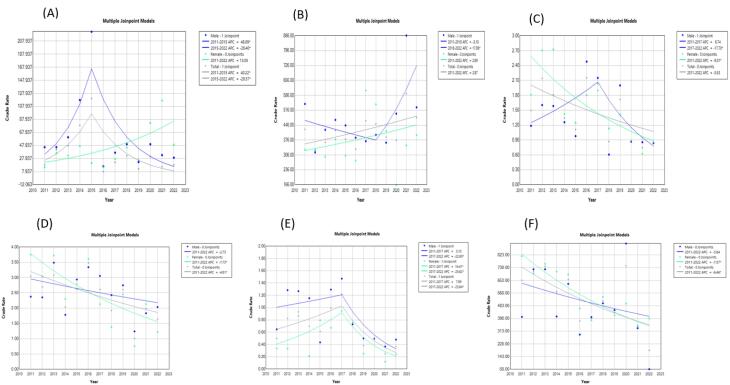
(A) Joinpoint models for co-infection rate of TB and AIDS, (B) Joinpoint models for the detection rate of SPPT with a smear grade of 3 + , (C) Joinpoint models for the incidence rate of EPT, (D) Joinpoint models for the incidence of SPPT, (E) Joinpoint models for the incidence rate of SNPT, (F) Joinpoint models for smear conversion rate two months after the treatment initiation. Abbreviations: SPPT: Smear-positive pulmonary tuberculosis; EPT: Extrapulmonary tuberculosis; SNPT: Smear-negative pulmonary tuberculosis

**Figure 2:**
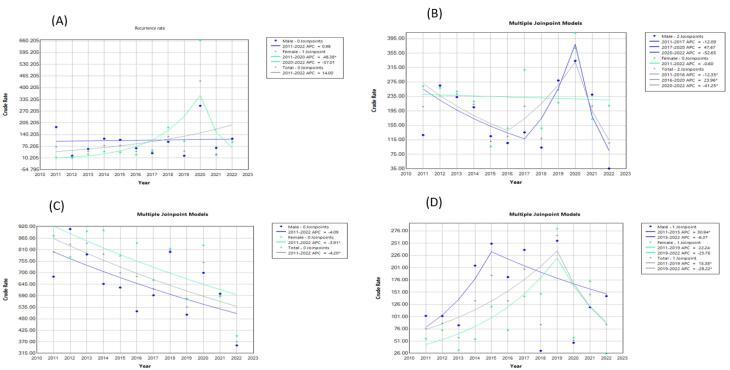
(A) Joinpoint models for recurrence rate, (B) Joinpoint models for TB detection rate by the private sector, (C) Joinpoint models for treatment success rate, (D) Joinpoint models for death rate from TB

 A significantly decreasing trend was observed in “the incidence of SPPT” index in females (AAPC: -7.72; 95% CI: -15.63, -1.10; *P* = 0.008) and overall population (AAPC: -4.8; 95% CI: -9.4, -0.81; *P* = 0.030) (Table 3). A significantly decreasing trend was observed in “the incidence rate of EPT” (AAPC: -9.30; 95% CI: -18.25, -2.03; *P* = 0.002) and “the negative rate of sputum smear two months after the treatment initiation” (AAPC: -7.57; 95% CI: -10.62, -5.34; *P* = 0.00007) in females. Additionally, “the treatment success rate” in both genders had a significantly decreasing trend. The “recurrence rate” in females had a rising trend in the mentioned time period (AAPC: 18.45; 95% CI: 3.23 to 46.47; *P* = 0.0002). The trend of the “co-infection rate of SPPT and HIV” index in 2011-2015 was increasing (APC: 40.21; 95% CI: 4.29 to 211.13; *P* = 0.03) and afterwards it was decreasing in both genders (APC: -28.57; 95% CI: -57.8, -18.14; *P* = 0.010), which was significant in males in 2015-2022 (APC-28.4046; 95% CI: -41.0052, -13.1126; *P* = 0.004). The changes in these indices are presented graphically in Figures 1 and 2.

**Table 3:** Trend of TB-related indicators by gender from 2011 to 2022

**Male**				**Female**				**Total**			
**Period**	**Change year**	**APC (95% CI)**	**AAPC (95% CI)**	**Period**	**Change year**	**APC (95% CI)**	**AAPC (95% CI)**	**Period**	**Change year**	**APC (95% CI)**	**AAPC (95% CI)**
The incidence of SPPT^a^											
2011-2022		-2.73 (-9.75,3.79)	-2.73 (-9.75,3.79)	2011-2022		-7.72 (-15.63, -1.10)	-7.72 (-15.63, -1.10)	2011-2022		-4.80 (-9.40, -0.81)	-4.80 (-9.40, -0.81)
The incidence rate of EPT^a^											
2011-2022			-4.19 (-15.39,5.73)	2011-2022		-9.30 (-18.25, -2.03)	-9.30 (-18.25, -2.03)	2011-2022		-5.52 (-12.38,0.56)	-5.52 (-12.38,0.56)
2011-2017	2017	8.73 (-3.45,87.98)									
2017-2022		-17.70 (-60.54, -3.67)									
The incidence of SNPT^a^											
2011-2022			-9.65 (-23.71,2.06)	2011-2022			-5.92 (-20.51,7.58)	2011-2022			-7.75 (-22.08,5.03)
2011-2017	2017	3.09 (-14.96,110.09)		2011-2017	2017	14.41 (0.52,115.54)		2011-2017	2017	7.99 (-3.09,115.44)	
2017-2022		-22.90 (-72.21, -3.71)		2017-2022		-25.62 (-68.03, -10.3)		2017-2022		-23.6 (-70.9, -10.26)	
The negative rate of smear 2 months after the treatment^b^											
2011-2022		-3.83 (-15.94,6.71)	-3.83 (-15.94,6.71)	2011-2022		-7.57 (-10.62, -5.3)	-7.57 (-10.62, -5.34)	2011-2022		-6.45 (-12.96, -1.49)	-6.45 (-12.96, -1.49)
The detection rate of SPPT with a smear grade of 3 +^b^											
2011-2022			4.06 (-1.50,9.17)	2011-2022		2.88 (-3.05,8.27)	2.88 (-3.05,8.27)	2011-2022		2.87 (-1.04,6.53)	2.87 (-1.04,6.53)
2011-2018	2018	-3.09 (-25.86,21.49)									
2018-2022		17.88 (0.10,68.50)									
Co-infection rate of HIV/TB^b^											
2011-2022			-6.74 (-23.07,7.80)	2011-2022		13.04 (-1.78,30.18)	13.04 (3.18,23.84)	2011-2022		-8.71 (-25.68,5.03)	-8.71 (-25.68,5.03)
2011-2015	2015	48.09 (16.49,226.46)						2011-2015	2015	40.21 (9.29, 211.13)	
2015-2022		-28.40 (-55.40, -18.43)						2015-2022		-28.57 (-57.8, -18.14)	
Treatment success rate^b^											
2011-2022		-4.08 (-10.73,1.72)	-4.08 (-7.71,-0.31)	2011-2022		-3.90 (-8.87, -0.09)	-3.90 (-8.87, -0.09)	2011-2022		-4.19 (-7.24, -1.73)	-4.19 (-7.24, -1.73)
TB detection rate by the private sector^c^											
2011-2022			-9.50 (-26.93,11.52)	2011-2022		-0.59 (-7.53,4.93)	-0.59 (-7.53,4.93)	2011-2022			-7.54 (-17.12, -1.56)
2011-2017	2017	-12.08 (-48.78,64.31)								-12.35 (-35.52, -0.66)	
2017-2020	2020	47.66 (-25.39,103.26)								23.95 (3.2683,58.4943)	
2020-2022		-52.65 (-85.10,37.94)								-41.24 (-68.8297, -6.1347)	
Recurrence rate^b^											
2011-2022		0.98 (-12.32,14.90)	0.98 (-12.32,14.90)	2011-2022			18.45 (3.23,46.47)	2011-2022		13.99 (-4.17,37.78)	13.99 (-4.17,37.78)
				2011-2019	2019	48.37 (32.47,119.70)					
				2019-2022		-57.01 (-80.06,10.55)					
The death rate from TB¥											
2011-2022			5.77 (-9.06,20.73)	2011-2022			6.69 (-21.76,41.53)	2011-2022			1.37 (-8.87,11.87)
2011-2015	2015	30.93 (7.26,211.02)		2011-2019	2019	22.23 (-25.02,399.81)		2011-2019	2019	15.38 (8.06,48.84)	
2015-2022		-6.36 (-51.97,2.63)		2019-2022		-25.76 (-89.89,50.36)		2019-2022		-28.22 (-67.63, -0.73)	

SPPT: Smear-positive pulmonary tuberculosis; EPT: Extrapulmonary tuberculosis; SNPT: Smear-negative pulmonary tuberculosis; APC: Annual percentage change; CI, Confidence interval; AAPC, Average annual percentage change; CI, Confidence Interval. ^a^ Per 100­000 population; ^b^ Per 100 SPPT patients; ^c^ Per 100 all TB types.

## Discussion

 In this study, we examined 10 selected TB indices separately in males, females, and the total population from 2011 to 2022. One of the key points emphasized in this study is the remarkable changes in the TB-related indicators over the years under investigation.

 Regarding the “co-infection rate of TB and HIV”, the trend in males has shifted from an increasing trend with a steep slope in 2015 to a decreasing trend, while a noticeable upward trend was observed in females. These changes may be due to various factors such as the prevalence of AIDS, differences in lifestyle patterns, and gender disparities in the supply and demand for health services. The reduction in the co-infection rate of TB and HIV may indicate successful efforts in the prevention and treatment of both diseases, demonstrating that continuous programs for controlling both diseases together have been effective. The rate of increase in viral load over time is higher in females than in males and females living with HIV have limited access to care and treatment in several countries.^9,10^ Additionally, in the study by Wang et al, the ASR (age-standardized rate) of HIV-XDR-TB (HIV-associated extensively drug-resistant tuberculosis) increased significantly by an average of 14.77% (95% CI: 11.05%, 18.62%) globally during 1990-2019, as indicated by the results of the Global Burden of Disease Study in 2019, while ASRs of HIV-DS-TB (HIV-associated drug-susceptible tuberculosis) and HIV-MDR-TB (HIV-associated multi-drug resistant tuberculosis) decreased after 2005.^11^ In the study by Osei et al, the prevalence of TB/HIV co-infection did not change significantly from 2013 to 2017.^12^ In the study by Wang et al, the age-standardized incidence rate and age-standardized prevalence rate of HIV and DS-TB co-infection exhibited an overall increasing trend from 1990 to 2019, and the prediction indicated a slow downward trend from 2019 to 2040.^13^ In the study by Mehri et al, the percentage of TB patients with known HIV status during 2003-2017 had an increasing trend in all World Health Organization (WHO) regions. The HIV/TB co-infection showed a decreasing tendency in all regions, except for Europe. Furthermore, the trend of HIV/TB co-infection in Europe was increasing during the studied period.^14^ The study by Salisu et al reviewed records of patients retrospectively from January 1, 2009, to December 31, 2018, in Suhum Municipal. A sharp increase in the trend of the co-infection was observed from 6 (14.6%) in 2009 to 21 (36.8%) in 2010. The highest co-infection prevalence (40.4%) was recorded in 2011. The study recorded an overall decreasing trend of the co-infection, and case detection rate for HIV among persons living with TB was high.^15^

 Regarding the “the incidence rate of EPT” index, there was a pattern of initially increasing and then decreasing trends in males, while in females, a consistently decreasing pattern was observed. The reduction in the incidence rate of EPTB may result from increased public awareness about this type of disease, as well as advancements in its diagnosis and treatment.^16^ In the study by Ben Ayed et al, the annual incidence rate of TB from 1995 to 2016 was 91.13 per 100 000 population. EPT was present, with an APC of 2.04.^17^ In the study by Fallah et al, it was observed that EPT had a decreasing trend from 2015 to 2019 in Iran^18^ which is consistent with our results.

 The increase in “the incidence rate of SPPT” in males from 2011 to 2022 may be related to factors such as reduced access to healthcare services, non-adherence to treatment regimens, and the spread of associated infections. This issue requires more precise planning for TB control and prevention. However, this indicator only measures part of the TB situation and does not reflect the frequency of individuals’ exposure to TB. In a study by Tao et al, it was shown that the overall incidence of PTB in children (7.62 to 3.74 per 100­000) decreased during 2005-2017, with a non-significant APC after 2010.^19^ In a study by Essien, the incidence of TB was examined and compared in Canada and the United States from 1953 to 2015. The TB rates for both countries were retrieved from 1953 to 2015. The AAPC change in Canada from 1975 to 2015 was -2.9%, compared to -4.1% in the United States.^20^ In a study performed by Brito et al on TB in Northeast Brazil (2001-2016), the overall incidence rate decreased from 44.84/100 000 persons in 2001 to 30.92/100 000 persons in 2016 (AAPC: -2.3; *P* < 0.001) ^21^. In a study conducted by Alavi et al in Khuzestan province, the results of evaluating the trend of TB from 2010 to 2019 showed no significant change in the overall TB trend, with an annual increase of 0.84% (95% CI: -5.17, 6.82). Additionally, the findings of regression analysis revealed a breakpoint indicating an annual increase of 18.10% (95% CI: 8.78 to 34.89) in TB incidence between 2010 and 2013, and an annual increase of 5.42% (95% CI: -10.04, -2.22) in incidence between 2013 and 2019. Moreover, from 2010 to 2012, an annual increase of 33.10% (95% CI: 15.77 to 48.06) in TB incidence was observed in males, while an annual decrease of 9.47% (95% CI: -14.02, -6.33) was observed in females.^22^ In a study by Lima et al on TB cases in Brazil from 2001 to 2016, an increasing trend in TB cases was observed in patients under 20 years old and 20-39 years old, especially in males,^23^ which is similar to the results of our study.

 The analysis of “The Incidence rate of SNPT” index, highlights the importance of early and accurate diagnosis of TB. The increase in SNPT cases may stem from increased public awareness and the development of advanced diagnostic methods. The rise in the frequency of this indicator could signify an increase in accurate TB testing and improvement in prevention programs. In the study by Tao et al, which investigated the epidemiological characteristics of SNPT in children in Shandong, China, from 2005 to 2017, it was demonstrated that the incidence of SNPT significantly increased.^19^ We also observed an increasing trend until 2017.

 «The negative rate of smear 2 months after the treatment initiation « may indicate treatment failure or non-adherence to it. This indicator highlights the need for careful monitoring and improvement of the treatment process. An increase in the percentage of negative test results after two months can indicate the effectiveness of treatment and prevent the spread of the disease. The bacilli load usually decreases steadily during treatment and is expected to be negative in most cases by the end of the second month of treatment, when the intensive phase of treatment ends.^24^

 «The detection rate of SPPT with a smear grade of 3 + », defined as the number of individuals diagnosed with TB who have a high smear grading of 3 + , serves as a measure for assessing disease severity and treatment follow-up. This information can help optimize public health programs for TB control and prevention. In the case of males, distinct periods of both decline and increase were observed. Higher sputum smear grade was significantly associated with the number of previous TB treatments, body mass index, and patient’s educational status in the multivariable ordinary logistic regression analysis.^25^ The probability of having a high grade of sputum smear among patients who had a history of two or more previous TB treatments is almost twice that of their counterparts.^26^. In females, a milder upward trend was observed, which may be attributed to disparities in access to healthcare services, increased awareness, and diagnostic accuracy.

 «The treatment success rate» shows a significant stability and continuous decrease, which is evident in both females and males. However, the limitation of this indicator lies in its failure to account for factors that may affect treatment efficacy, such as drug resistance, malnutrition, and so forth ^27^. The WHO report of 2020 shows that the global treatment success rate of new cases of TB was 85% and 76% for TB patients living with HIV^28^ and the TB success rate was found to be 78.9% in Africa and 80.1% globally.^29^ An epidemiological study conducted by Aketi et al on pediatric TB in SSA reported a treatment success rate of 69.6%.^30^ In a study by Tao et al in Shandong, China, an overall treatment success rate of 94.2% was achieved among pediatric TB cases from 2005 to 2017.^19^ The disparity in treatment outcomes between these studies may be due to differences in the study population’s age, gender, and disease severity, the existence of comorbid conditions, tobacco use, the drug resistance pattern, social determinants of health, and socioeconomic characteristics.^31^

 «TB detection rate by the private sector» was one of the key findings of this study, showing significant changes in TB incidence over the years. In this indicator, a decreasing trend with a mild slope was observed in females and in males; in other words, three distinct periods with different decreasing, increasing, and again decreasing trends were observed, which may indicate fluctuations in the performance of the private sector and changes in policies or treatment protocols. The private sector plays an important role in TB elimination by providing access to quality TB care services.^32^ In the study by Khan et al, it was found that novel approaches to TB case-finding involving the private sector can substantially increase case notification. In the intervention area, TB case notification to the private sector increased two times (from 1569 to 3140 cases) from 2010 to 2011, a 2.21 times increase (95% CI 1.93, 2.53).^33^ The increase in the detection rate by the private sector can indicate the effective role of this sector in controlling TB incidence. In the study by Kumar et al, the annual new AFB-positive case notification rate increased by 21%, from 27.8/100 000 in 2000 to 33.5/100 000 in 2002. This public-private partnership substantially increased TB case detection and established a sustainable framework for private sector involvement in TB control. In the setting of a strong public sector program, the combination of active surveillance of private laboratories along with physician sensitization is a promising approach to improve TB case detection.^34^

 Regarding «the recurrence rate» index, it was observed that during the study period, males experienced a very slight increase. On the other hand, females showed a clear trend toward an increase, which may be the result of changes in environmental or biological factors. However, the recurrence rate decreased relatively rapidly at the end of the period, which is likely indicative of the implementation of effective prevention and treatment programs or changes in environmental and social factors. In a study by Pascopella et al, out of 23 517 TB patients, 148 individuals (0.63%) had delayed recurrence.^35^ Furthermore, in a study by Tao et al, the recurrence cases decreased by approximately 80% (from 5.11 to 1.01 per 100 000) from 2005 to 2017.^36^ In our study, relapse cases decreased by 35.29% (from 7.27 to 11.11 per 1000).

 In the indicator of «The death rate from TB», initially, an increase in the rate of mortality from TB was observed in both genders, which could be due to factors such as increased disease distribution or fluctuations in the quality of care and treatment. However, then a decrease in the mortality rate occurred, which could be attributed to improvements in treatment modalities, advancements in prevention, effective public health interventions, and more importantly, the COVID-19 pandemic.^37^ The risk of death due to TB is higher in males than in females and this can be due to systemic factors.^38^. In the study conducted by Liaw et al in Sabah on 10 058 TB patients, there were 842 deaths (9.2%) ^39^. In our study, female patients had a sharper decline than male patients. In the study conducted by Dhamnetiya et al in India over the past three decades (from 1990 to 2019), the standardized incidence and mortality rate of TB in India decreased from 121.72 to 36.11 per 100 000 population. The decreasing trend was more prominent in females (AAPC: -4.35; 95% CI: -5.12, -3.57; *P* < 0.001) compared to males (AAPC: -3.11; 95% CI: -3.74, -2.48; *P* < 0.001). For specific age groups, the incidence and mortality rate of TB decreased in both males and females across all ages during this period.^40^. Additionally, in the study by Varshney et al, there was a significant reduction in TB cases and total deaths across India since the emergence of COVID-19, which is consistent with our study.^41^

 However, it is essential to acknowledge the limitations of this study. In this study, we only considered data from 2011 to 2022. This might overlook long-term changes and trends, with the possibility of significant changes in previous or subsequent years. Additionally, variables such as economic status, education level, history of other diseases, and access to healthcare services, which may influence TB status, were not controlled in this study. Despite these limitations, this study remains valuable as it provides us with information that can aid in improving TB prevention, diagnosis, and treatment strategies and offers crucial insights into the occurrence and other indicators of TB in Hamadan.

**Highlights**

- Significant decreasing trends were observed in TB incidence rates among smear-positive female patients.
- Significant decreasing trends were observed in extra-pulmonary TB rates among males and females.
- Significant decreasing trends were observed in TB treatment success rates among both genders.
- The recurrence rate among females exhibited a notable upward trend.

## Conclusion

 The results of this study showed that the state of TB has had a relatively successful trend in some of the studied indicators since 2011, and we have seen a decrease in the co-infection of TB and AIDS, SPPT, and EPT in the region. However, an increase in mortality due to TB was observed, especially in females. Moreover, a decrease in the success rate of treatment was observed in females. It can be concluded that the patterns of changing indicators are different based on gender; therefore, the need for gender-based approaches in the management and prevention of TB is felt. This data analysis provides valuable information about the state of TB in Hamadan city, which can help to improve programs for the prevention, treatment, and control of TB in this community. In general, the results of the study show that despite the progress made in the field of TB control, there is still a need for careful attention and planning to reduce the incidence of TB and improve public health conditions.

## Acknowledgments

 This study is a part of thesis for the MSc student of Epidemiology. The study was approved by the Research Vice-Chancellor of Hamadan University of Medical Sciences (140302251370). The authors would like to express their thanks to all those who contributed to the study.

## Competing Interests

 None.

## Ethical Approval

 This study was approved by the Ethics Committee of Hamadan University of Medical Sciences (IR.UMSHA.REC.1403.123).

## Funding

 None.

## Supplementary files

Supplementary file 1 contains Table S1.
