# Supplementary material for: Changes in The Trends of Tuberculosis-related Indicators in Hamadan Province Using the Join Point Regression Approach From 2011 to 2022
Source: J Res Health Sci. 2024 Dec 25;25(1):e00641. doi: 10.34172/jrhs.2025.176 (PMC11833495; doi:10.34172/jrhs.2025.176)
Supplement: Supplementary file 1 — contains Table S1. [file jrhs-25-e00641-s001.pdf]

JRHS
Journal of Research in Health Sciences
doi:10.34172/jrhs.2025.176
JRHS 2025; 25(1):e00641

Supplementary file 1

Table S1. Formulas for estimating the specified indicators

Index	Formula
Incidence rate of smear-positive pulmonary TB (SPPT)	Number of new smear-positive pulmonary TB cases reported per 100000 population at risk during a specified time period
Incidence rate of extra-pulmonary TB (EPT)	Number of new EPT cases reported per 100000 population at risk during a specified time period
Incidence rate of smear-negative pulmonary TB (SNPT)	Number of new SNPT cases reported per 100000 population at risk during a specified time period
Co-infection with AIDS	Number of TB cases with HIV co-infection per total number of SPPT cases reported during a specified time period
Relapse rate	Number of patients who relapse after treatment per total number of SPPT cases during a specified time period
Smear conversion rate (two months after treatment initiation)	Number of patients with negative smears at two months per total number of patients with positive smears at treatment initiation during a specified time period
TB death rate	Number of deaths due to TB per total number of TB cases reported during a specified time period
Diagnosis rate of pulmonary TB with a smear grade of 3+	Number of pulmonary TB cases with a smear grade of 3+ per total number of SPPT cases diagnosed during a specified time period
Treatment success rate	Number of patients successfully treated per total number of SPPT patients treated during a specified time period
TB diagnosis rate by the private sector	Number of TB cases diagnosed by private healthcare providers per total number of TB cases reported during a specified time period
